# Spatial–Temporal Hotspot Management of Photovoltaic Modules Based on Fiber Bragg Grating Sensor Arrays

**DOI:** 10.3390/s25154879

**Published:** 2025-08-07

**Authors:** Haotian Ding, Rui Guo, Huan Xing, Yu Chen, Jiajun He, Junxian Luo, Maojie Chen, Ye Chen, Shaochun Tang, Fei Xu

**Affiliations:** 1 Key National Laboratory of Solid State Microstructures, Collaborative Innovation Center of Advanced Microstructures, College of Engineering and Applied Sciences, Nanjing University, Nanjing 210023, China; haotianding@smail.nju.edu.cn (H.D.); balance.rg@smail.nju.edu.cn (R.G.); 602023340020@smail.nju.edu.cn (H.X.); chenyu1997c@163.com (Y.C.); jiajunhe@smail.nju.edu.cn (J.H.); dg21220040@smail.nju.edu.cn (J.L.); chenmaojie@smail.nju.edu.cn (M.C.); 2 College of Physics, Nanjing University of Aeronautics and Astronautics, Nanjing 210016, China; 3 Haian Institute of High-Tech Research, Nanjing University, Nanjing 226600, China

**Keywords:** hot spot management, photovoltaic module, fiber Bragg grating temperature sensor, machine learning, cooling hydrogels

## Abstract

Against the backdrop of an urgent energy crisis, solar energy has attracted sufficient attention as one of the most inexhaustible and friendly types of environmental energy. Faced with long service and harsh environment, the poor performance ratios of photovoltaic arrays and safety hazards are frequently boosted worldwide. In particular, the hot spot effect plays a vital role in weakening the power generation performance and reduces the lifetime of photovoltaic (PV) modules. Here, our research reports a spatial–temporal hot spot management system integrated with fiber Bragg grating (FBG) temperature sensor arrays and cooling hydrogels. Through finite element simulations and indoor experiments in laboratory conditions, a superior cooling effect of hydrogels and photoelectric conversion efficiency improvement have been demonstrated. On this basis, field tests were carried out in which the FBG arrays detected the surface temperature of the PV module first, and then a classifier based on an optimized artificial neural network (ANN) recognized hot spots with an accuracy of 99.1%. The implementation of cooling hydrogels as a feedback mechanism achieved a 7.7 °C reduction in temperature, resulting in a 5.6% enhancement in power generation efficiency. The proposed strategy offers valuable insights for conducting predictive maintenance of PV power plants in the case of hot spots.

## 1. Introduction

With the globalized energy crisis and increased atmospheric pollution, traditional fossil fuel power is faced with the gradual depletion of resources and an increasing risk to the environment. Recently, solar energy has become a focus of attention, with the advantages of being sustainable, clean, renewable, and inexpensive [1,2]. PV modules convert solar energy directly into electricity and are the basic application of solar energy [3]. As PV panels are in service for long time and in poor environments, lower performance ratios and safety risks occur frequently under real-world conditions [4,5,6]. The primary concern is the hotspot effect of PV modules caused by obscured shadows [7], breakages and defects [8], animal traces [9], uneven concentration of light, [10] and aging [11]. The hotspot phenomenon in PV modules can lead to rapid aging or unexpected failure along with potential fire hazards [12]. It is worth pointing out that the output power is suppressed by hotspots, causing the economic loss of PV power plants [13]. In addition, the most serious complication is the extension from the cell with a hotspot to surrounding cells, resulting in an irreversible damage to the modules. Therefore, the hotspot issue has attracted adequate attention from researchers, who study the mechanisms and effects in depth and develop effective solutions with regard to detection, diagnosis and cooling.

Analyzing and resolving the hotspot effect of PV modules is generally divided into a two-step process, such as the detection and cooling of the hotspot region. For the accurate detection of PV module temperature field and hotspots, the methods can be divided into two categories: the electrical characteristics method and the thermal characteristics method. The electrical characteristics method often uses the IV curve, impedance, and other parameters of the PV module for the detection of hotspot conditions. Ma et al. placed the types of hotspot module into three categories using the IV curve of PV modules [14]. Kim et al. observed the significant changes in the parallel capacitance and parallel resistance when a hotspot occurred [15]. For PV plants, the electrical characteristic methods for the detection of hotspots are an expensive, vague, and inefficient. The methods based on thermal characteristics detect hotspots by monitoring the temperature distribution of the PV panels. Bohorquez et al. made use of digital temperature sensors installed on the back of the PV panel for measuring the temperature after calibration [16]. Although the electrical sensor has high-precision measurement and is inexpensive, the surface temperature of the PV panels cannot be measured due to the shadow of the sensor and plenty of sensors need to be arranged for PV power stations [17]. Thermographic analysis is the most widely accepted assessment method for identifying hotspots in PV systems [18,19,20]. Through the image-processing algorithm, the collected infrared thermal images of PV panels are analyzed to look for cells with the hotspot effect. However, the accuracy of thermography in measuring PV panel temperature is heavily influenced by environmental and climatic factors. Additionally, the characteristics of high costs, complex processing flows and low detection efficiency prevent the thermal image method from monitoring distributed hotspots of the PV array in real-time.

For the second-step process, current cooling strategies of PV modules include two types: active cooling and passive cooling. Active cooling systems involve the adoption of a coolant, such as air or water, to effectively remove heat from the PV panel surface [21,22]. This method has been broadly acknowledged to exhibit high cooling efficiency, but it requires a wide installation space and has a complicated structural design; it also consumes extra power when operating the cooling equipment and circulating the coolant [23]. Firoozzadeh, Shiravi, and Shafiee used aluminum fins and pasted them on the backside of the PV panel with the help of the thermal conducting paste [24]. The temperature reduction in the PV panel reached levels of 7.4 °C from 85 °C, and this reduction led to increase in efficiency. Air cooling employs air as a coolant and dissipates heat using the heat convection approach to reduce the temperature of PV panel, but the deployment of an oversized heatsink significantly affects the space utilization of the PV power plant and unstable airflow results in temperature fluctuations between PV modules [25]. With regard to the liquid cooling system, a heat pipe comprises three parts: an evaporator, an adiabatic transfer section, and a condenser with working fluid inside in the liquid phase as a common boundary between two things. Alizadeh et al. used pulsating heat pipes as they are compact with high thermal efficiency due to the high amplitude oscillation in gas bubbles and liquid column penetration in both the condenser and evaporator [26]. The temperature reduction in the PV panel reached values of 16.1 °C from 51 °C, and this reduction led to an 18% increase in efficiency. Coolant liquids such as water and ethanol can continuously control the PV panel temperature, while a large quantity of liquid is consumed and wasted during cooling. On the other hand, passive cooling derives from natural convection and radiative cooling [27,28,29]. In the case of radiative cooling, it provides a relatively low cooling power of 40~120 W/m^2^ only under sunny weather in spite of low power consumption [30].

With the development of advanced sensing technology and multifunctional materials, a potential effective and cheap hotspot management system can be explored and optimized for widespread application in PV power stations. Compared with digital temperature sensors and infrared thermal images, the FBG sensor can achieve the temperature distribution with small-sized, anti-electromagnetic interference, high detection efficiency and accuracy [31,32,33]. Therefore, FBG sensors are ideally suited to measuring the temperature field and detecting hotspots in PV panels [34,35]. However, the existing studies have not addressed the categories of hotspots, the suppression of hotspots, or the impact of hotspots on the power generation performance of PV panels. To solve these problems, we combine the FBG sensor array for hotspot diagnosis based on ANN with cooling hydrogels to develop a hotspot management strategy of PV modules. The hygroscopic hydrogel can absorb atmospheric water vapor based on sorption in the evening and release the stored water for cooling during the day [36,37,38]. On this basis, field tests are carried out, in which the FBG temperature sensor arrays identify the hotspot region and temperature of the PV module, and then a classifier based on an optimized ANN distinguishes hotspots with accuracy of 99.1%. Using cooling hydrogels as feedback can realize a decrease in temperature of 7.7 °C and an increase in the power generation efficiency of the PV module up to 5.6%. Moreover, the time spent on the occurrence and cooling of hotspots has contributed to a deep understanding of opto-electro-thermal coupled mechanisms in PV modules. The results offer valuable insights for conducting the predictive maintenance of PV power plants in the event of hotspots.

## 2. Result and Discussion

### 2.1. The Principle of Hotspot Management for PV Modules

**Figure 1:**
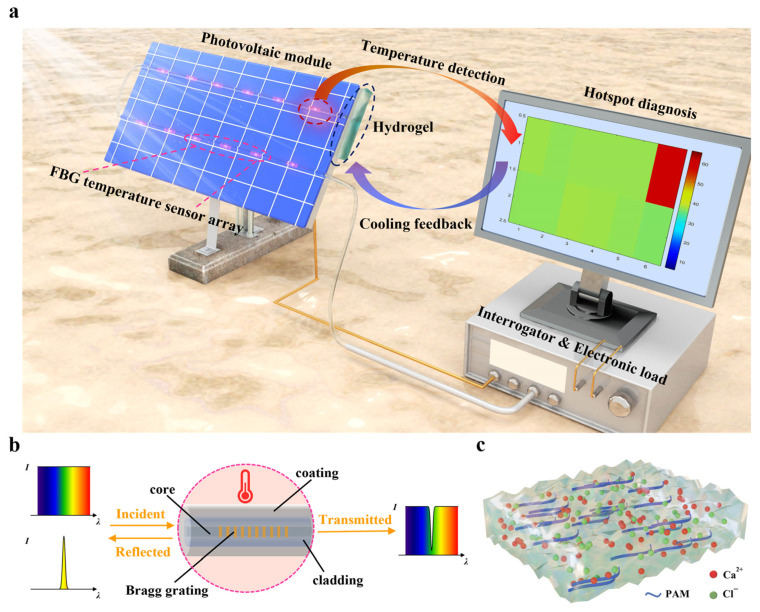
A schematic of the PV module attached with the hotspot management system and evaluation of cell performance with optical fiber attached. (**a**) The schematic diagram of the hotspot management system for a solar cell integrated with the FBG temperature sensor array and the PAM-CaCl_2_ hydrogel. (**b**) The schematic expression of the structure of FBG. (**c**) Schematic of the hygroscopic hydrogel applied in this work, with a PAM hydrogel as the substrate and PAM-CaCl_2_ as the sorbent.

The defective regions of a PV module (obscured, cracked, delaminated, bubbled, etc.) [39] are regarded as loads that consume energy generated from other regions. This situation leads to the local overheating of the PV module, a phenomenon known as the “hotspot effect” has occurred, as shown in Figure 1a. The hotspot effect plays an important role in reducing power generation efficiency and the life span of PV modules in the process of power generation. In this study, a hotspot management strategy on the spatial–temporal scale is proposed; the FBG temperature sensor array is employed for hotspot detection and polyacrylamide (PAM)/CaCl_2_ hygroscopic hydrogel is used as a cooling layer. The proposed thermal management system applied to the PV panel consists of two temperature sensor arrays with 12 FBGs, a commercial interrogator, a computer equipped with an optimized ANN classifier and a piece of hydrogel. Every item used is described in detail in the following section and in the Supporting Information. Moreover, the direct current (DC) electronic load is adopted to study the effects of localized hotspots on the cell performance.

There are several types of fiber-optic sensors that can be used for temperature sensing, including FBG [40], a fiber Fabry Pérot interferometer [41], long-period fiber grating [42], and distributed fiber-optic sensing [43]. Among the fiber optic temperature sensors, FBG sensors are the most commonly fabricated and demodulated. Due to the high-capacity performance of optical multiplexing technology, the FBG sensor arrays are widely used in various engineering applications. The FBG is essentially an optical filter that exists in the core of an optical fiber waveguide. The Bragg grating region has a periodic refractive index modulation along the fiber core, as shown in Figure 1b. The FBG structure was fabricated using femtosecond laser direct writing (see Note S1 for details). In particular, the reflected light of an FBG structure satisfies a narrow Bragg resonance from broadband incident light. The central wavelength of the reflected light corresponds with the formula [44]:

(1) $$\lambda_{B}=2n_{\mathrm{eff}}\Lambda$$

where *n*_eff_ denotes the core effective refractive index and *Λ* represents the period of grating. The Bragg wavelength shift occurs due to thermal expansion and the thermo-optic effect from temperature variations. As a result, the relationship between the wavelength shift in the FBG resonance and temperature is presented by the following equation [45]:

(2) $$\Delta \lambda_{B}=\left(\alpha_{0}+\beta_{0}\right)\cdot \lambda_{B}\cdot \Delta T$$

where *α*_0_ is the thermal expansion coefficient and *β*_0_ is the thermo-optic coefficient of the optical fiber. It can be concluded that there is a linear relationship between the wavelength shift in the reflected resonance and the temperature change and the temperature can be measured by tracking the fiber Bragg wavelength shift.

On the other hand, FBG is sensitive not only to the temperature but also to the strain. Consequently, the influence of strain needs to be considered. The FBG arrays were attached to the surface of a PV panel with thermal conductive silicone paste (Figure S1). The thermal paste can keep the grease state at −50~230 °C without curing or adhesive force. The internal stress of thermal paste has essentially no influence on the Bragg wavelength of the FBG sensor over the temperature range of the PV panel. Moreover, the thermal conductive silicone grease can prevent uneven heat transport of the FBG. In order to realize hotspot detection of the PV panel, the reliability of the FBG sensor for temperature monitoring needs to be proven. Through marking the PV panel temperature measured point in the thermal imaging screen, the measured area of the FBG temperature sensor can be matched to the thermal imaging measured point. As a result, the real-time temperature measurement of the FBG sensor was consistent with the infrared thermal imaging result (Figure S2). Specifically, the comparison of distributed temperature detection with two temperature sensing methods for the whole PV panel is presented in the section of hotspot diagnosis for PV panels.

A high temperature in the PV panel not only causes safety hazards such as shortened PV cell life due to the hotspot effect but also reduces power generation efficiency [46]. To decrease the temperature of a PV panel, an effective cooling technology needs to be considered. In recent years, a new cooling strategy using the hygroscopic hydrogel has been proposed [47,48]. In this study, the PAM-CaCl_2_ hygroscopic hydrogel is applied to cool the panel down; it is attached on the backside of the hotspot zone in the PV panel (Figure S3). A schematic illustration of the hygroscopic hydrogel is given in Figure 1c. The hydrogel including hygroscopic salts is composed of polyacrylamide (PAM) as the substrate and calcium chloride (CaCl_2_) as the sorbent material. The PAM hydrogel acts as a porous and hydrophilic structure and SEM images of the hydrogel framework are shown in Figure S4, promoting the adsorption and diffusion of water and loading some functional materials. Although the PAM hydrogel can store a lot of water, it is lost quickly when exposed to outdoor conditions. Therefore, it is necessary to employ an adsorbent material such as metal–organic frameworks or hygroscopic salts [49,50]. After immersing the PAM dried hydrogel in a highly concentrated CaCl_2_ solution, the PAM-CaCl_2_ exhibits a higher water adsorption capacity and can recover by adsorbing atmospheric water vapor at night (see Note S1 for details). The synthesized hydrogel behaves as a form of gel-like solid, adsorbing water vapor from the atmosphere during the night and evaporating the adsorbed water during the day through waste heat from hotspots in the PV panel. In this way, the local high temperature of a hotspot in the PV cell can be clearly reduced and the risk of fire can be prevented thanks to the cooling method using a PAM-CaCl_2_ hydrogel.

It is worth noting that the pasted FBG sensor has little impact on the electrochemical performance of a solar cell. In order to verify this view, a long-term open-circuit voltage test for the PV cell was designed under the condition of the normal radiation of one sun (1000 W/m^2^) and room temperature (25 °C). Through setting the power of the xenon lamp in the experiment, a specific and stable simulated sunlight was obtained. From the two close voltage time curves of solar cells shown in Figure S5, it is clear that the output voltage of both cells can rapidly decay from 3.77 V to 3.18 V within half an hour and stabilize for a long time. Comparing the voltage of solar cells with FBG and without FBG for 12 h, it is confirmed that the optical fiber has little influence on the electrochemical behavior of a solar cell.

### 2.2. Temperature Field Analysis of a PV Cell

**Figure 2:**
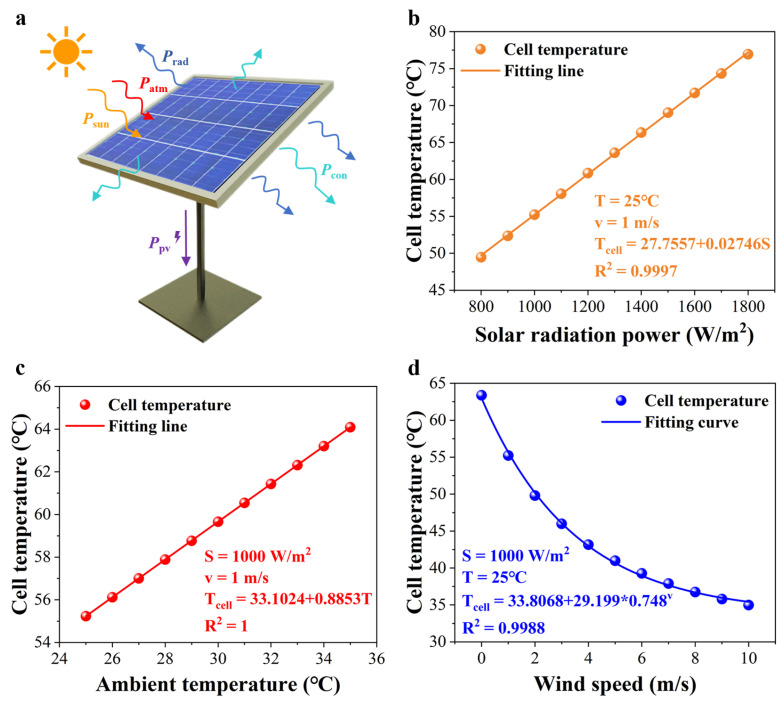
The temperature model of a PV cell. (**a**) A schematic illustration of the energy balance of a PV panel. (**b**) The relationship between solar radiation power and the cell temperature. (**c**) The relationship between ambient temperature and the cell temperature. (**d**) The relationship between wind speed and the cell temperature.

As illustrated in Figure 2a, a PV panel can absorb power from incident solar radiation and atmospheric radiation, consuming about 75% energy via thermal convection and thermal radiation into the environment [51]. Only a small part of the absorbed energy from a PV cell can converted into electricity [52]. In fact, most of the absorbed energy is stored as heat energy and leads to the increased PV cell temperatures. To avoid heat damage and improve the heat dissipation strategy, it is vital to study the temperature model and relevant influencing factors of a PV cell [53].

To build the opto-thermal coupled model of a PV cell, a simulator (COMSOL Multiphysics) was applied to solve the corresponding problem. As seen in Figure S6, the PV cell model is composed of several materials stacked in order from top to bottom including glass, a polycrystalline silicon solar cell, a TPT backsheet and air. Under the condition of one sun radiation, ambient temperature of 25 °C and natural thermal convection, the surface temperature simulation result of a PV cell was 57 °C (see Note S1 for details). Moreover, the temperature of a PV cell will rise up to 60 °C in the hot summer, causing irreversible damage. After that, the relationship between influencing factors and the cell temperature needs to be studied further. Figure 2b–d demonstrates that solar radiation power, ambient temperature, and wind speed have a significant influence on the cell temperature. In contrast to the previous PV module temperature prediction model [54], the relationship between wind speed and the cell temperature is not linear but exponential. The multiple fitting equation model is proposed in equation:

(3) $$T_{\mathrm{cell}}=0.02746S+0.8853T+29.199*0.748^{v}-15.7857$$

where *S* is the solar radiation power, *T* refers to the ambient temperature and *v* is the wind speed. Generally, considering the performance of thermal convection, a PV panel is placed with a tilt angle of 45° [55]. By monitoring the three parameters in real time, the PV cell temperature can be predicted and then abnormal temperatures caused by the hotspot effect in a PV panel can be detected as well. In addition, researchers can build a data model for PV power plants and predict the temperature of PV module by collecting environmental parameters in all seasons. Based on the above simulation model, a hydrogel module was added into the bottom of the PV cell model. Under the same conditions shown in Figure S5, the simulated surface temperature of a PV panel with a cooling hydrogel was 45.2 °C (Figure S7). The surface temperature difference between two PV cell models was 11.8 °C, which means that a hygroscopic hydrogel can realize efficient passive cooling. It is noted that the duration of cooling and the actual cooling effect need to be explored via outdoor tests. As a result, keeping the hotspot region at a low temperature with a cooling hydrogel can prevent a PV module from catching fire.

### 2.3. The Characterization of the Hotspot Management System

**Figure 3:**
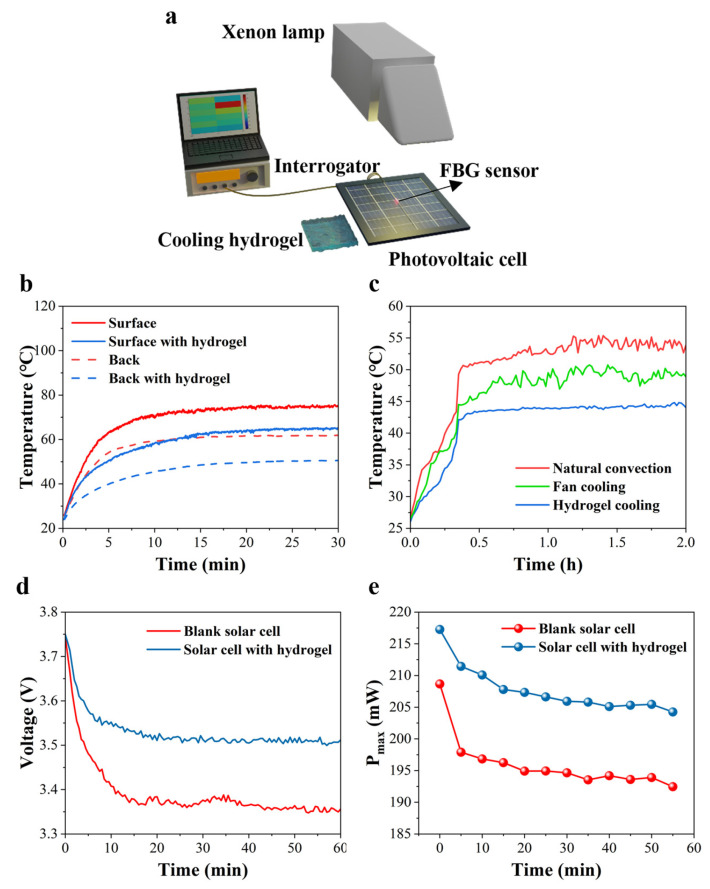
PV cell cooling performance and characteristics under simulated laboratory conditions. (**a**) Schematic diagram of the fiber-optic/hydrogel integrated temperature control system. (**b**) The surface and back temperature comparison between a bare PV cell and a PV cell with the cooling hydrogel. (**c**) The surface cooling effect of natural convection, fan and hydrogel. (**d**) V_oc_ and (**e**) P_max_ of PV cell with and without the hydrogel cooling layer.

One hotspot management system is the fiber-optic temperature sensor and cooling hydrogel integrated temperature control system, as presented in Figure 3a. A xenon lamp was used as simulated sunlight and change the sunlight irradiation was changed by setting the power. The FBG sensor was pasted on the surface of the PV cell and a commercial interrogator was used to demodulate the FBG signal and convert the central wavelength of FBG to temperature. In addition, the surface of the PV cell was cooled down using a PAM-CaCl_2_ hydrogel. To fully understand the temperature variations in the PV cell during the period of illumination, two FBG sensors were attached on the surface of a PV cell and the other on the back, and we monitored the temperature in real-time. First, the relationship between the central wavelength and temperature for two FBG sensors needs to be calibrated before a temperature test is conducted (see details in Note S1) [56]. As shown in Figure S8, the temperature variation for each FBG is nearly linear and the slopes of two fitting lines are similar. With the aid of a solar power meter, the solar power radiation of a xenon lamp can be determined by adjusting the output power. Figure 3b presents the surface and back temperature comparison between a bare PV cell and a PV cell with the cooling hydrogel. Like the regions in South Asia and Africa, the peak sunlight radiation can exceed 1.2 kW/m^2^ in the summer at midday [57]. Under the solar radiation of 1200 W/m^2^, the surface and back temperatures of a cell reached 74.8 °C and 61.7 °C, respectively. The two-side temperature drops of the PV cell were 10 °C and 11.3 °C when the cooling hydrogel was employed, as depicted in Figure S9. Furthermore, the temperature profiles of the PV cell under one sun irradiation with different cooling methods are shown in Figure 3c. Compared to fan cooling with a wind speed of 1 m/s, the hydrogel cooling effect is better and more stable. Specifically, the PAM-CaCl_2_ hydrogel cooled the surface of a cell by 9.4 °C instead of 4.6 °C when cooled using a fan, as demonstrated in Figure S10. The practical hydrogel cooling performance of the hydrogel is consistent with the previous simulated result. Surprisingly, the temperature fluctuation of the cell was 0.3 °C with hydrogel cooling, and less than 1.1 °C with the air cooling. This phenomenon can be interpreted based on the adaptive water evaporation of a PAM-CaCl_2_ hydrogel according to the environment, and it has the significant advantage of generating power for PV cells. As an active cooling strategy, air cooling demands more complex systems and greater investments [58]. Considering no extra energy output, the passive cooling with a hydrogel is more suitable. The cooling performance of the hydrogel can be affected by different environmental conditions, such as temperature and humidity. Accordingly, we tested the cooling performance under varying light conditions while keeping the ambient temperature and humidity in the laboratory constant. As shown in Figure S11, the cooling temperature of the photovoltaic panel under 0.8 sun it was 9.5 °C, under 1.0 sun was 12.7 °C, and under 1.2 sun it was 14.8 °C. Although the cooling effect of the hydrogels varies in different environments, they can reduce the high surface temperature of photovoltaic panels to a certain extent. As mentioned above, the wind speed also affects the temperature of the PV panels; the effects of varying wind speeds on the cooling performance of hydrogels are further discussed below. As shown in Figure S12, the maximum cooling temperature of the hydrogel reached to 12 °C at the wind speed of 2 m/s. It is worth noting the dual effect of wind speed on the cooling of the hydrogel. On the one hand, the appropriate wind speed can enhance the cooling effect; on the other hand, a wind speed that is too high may have adverse effects. Specifically, a high wind speed can cause the water in the hydrogel to be lost rapidly, so that the cooling effect of the hydrogels will be significantly reduced.

It is necessary to record the features of a PV cell under the test condition. The IV curves of a PV cell with the cooling hydrogel under 1 kW/m^2^ sunlight irradiation are shown in Figure S13b; the currents dropped with increased voltage. Accompanied with a rapid rise in cell temperature, the IV curves acquired after longer radiation period presented lower currents than those obtained after a shorter radiation time, implying poorer behaviors during longer periods of sunlight irradiation and a rapid decline in cell performance. However, the PV cell without the cooling hydrogel had worse power generation performance (Figure S13a). Obviously, when reaching the maximum of power with a similar current, the voltages of the PV cell with hydrogels were higher than those of the PV cell without hydrogel. Equally, the crucial characterizations were recorded, including the open-circuit voltage (V_oc_) and maximum power (P_max_) of the PV cell, as seen in Figure 3d,e. When the cell was operating without the PAM-CaCl_2_ hydrogel, the open-circuit voltage quickly decreased from 3.74 to 3.37 V within 20 min, followed by noticeable fluctuations. In contrast, the V_oc_ of the PV cell with the PAM-CaCl_2_ hydrogel dropped from 3.75 to 3.51 V by the end of the first 20 min, followed by a slow decrease to 3.5 V after one hour (Figure 3d). The V_oc_ increased from 3.39 V to 3.53 V by integrating a cooling hydrogel, as shown in Figure S14. The standard deviation of V_oc_ between a blank solar cell and a solar cell with the PAM-CaCl_2_ hydrogel was 0.06 V and 0.04 V, respectively. A comparable trend was clearly discovered for P_max_ of the PV cell, which allowed the cell with a cooling hydrogel to generate more power. The P_max_ of the PV cell without the cooling hydrogel quickly dropped from 209 to 198 mW within the first 5 min and decreased from 198 to 190.5 mW after 50 min, and the P_max_ of the PV cell cooled by the hydrogel slowly dropped from 217 to 211 mW in the first 5 min and decreased from 211 to 205.5 mW after 50 min (Figure 3e). The P_max_ was increased from 196 mW to 208 mW by integrating a cooling hydrogel. The standard deviation of P_max_ between a blank solar cell and a solar cell with the PAM-CaCl_2_ hydrogel were 4.3 mW and 3.7 mW, respectively. With the PAM-CaCl_2_ cooling layer, the P_max_ of the PV cell was promoted by 6.1%. The effective light-sensitive area for the solar cell used in the experiments was 16 cm^2^. Therefore, the overall system efficiency was raised from 12.2% to 13% under one sun irradiation.

The results above obviously show that the temperature of a solar cell can be reduced by the evaporation of water from the PAM-CaCl_2_ hydrogel, thus minimizing the influence of the temperature rising and improving the energy conversion performance. To better validate this view, the same experiment was conducted by using a flexible solar cell with copper indium gallium selenide, as displayed in Figure S15. The FBG sensor is also flexible and can be bendably pasted onto the flexible solar cell. The PAM-CaCl_2_ hydrogel achieved a cooling effect which was beyond 20 °C, and the V_oc_ of the flexible PV cell rapidly rose from 1.77 to 1.83 V within 3 min (Figure S15b). The reason for the fast-cooling effect and improved cell performance is that the flexible cell is very thin with the thickness of a few hundred microns, resulting in quick heat transfer. Therefore, the fiber-optic temperature sensor and cooling-hydrogel-integrated temperature control system not only detects the temperature profiles of the PV cell, but also releases the heat from a PV cell and improves the cell performance.

### 2.4. Hotspot Diagnosis for PV Panels

**Figure 4:**
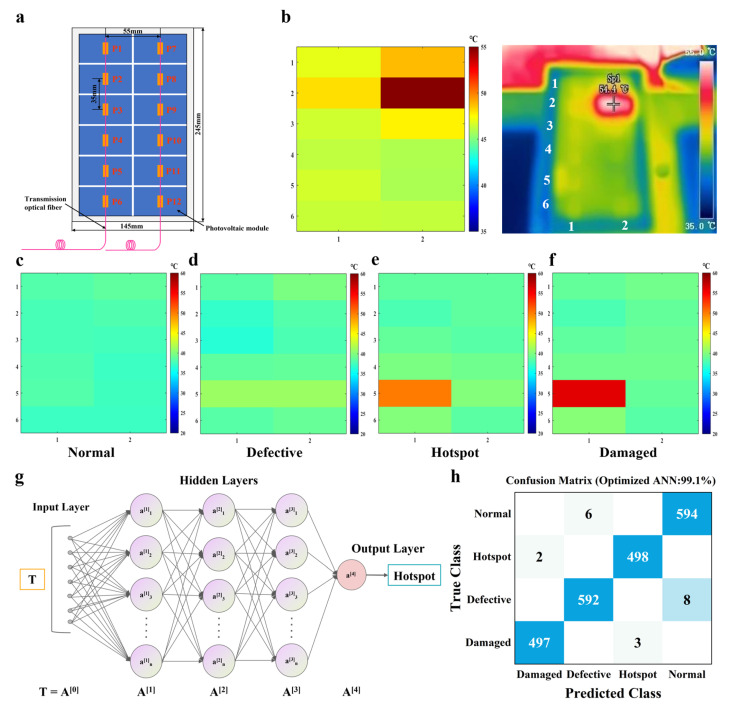
Outdoor hot spot detection and classification. (**a**) Structure diagram of the FBG sensor arrays pasted on the PV panel. (**b**) The hot map by FBG arrays and infrared thermal image of a hot spot on the PV panel. The hot map of (**c**) a normal PV panel, (**d**) a defective PV panel, (**e**) the PV panel with a hotspot and (**f**) a damaged PV panel. (**g**) Schematic expression of ANN with a Bayesian optimization for classifying PV panels. (**h**) Confusion matrix of the validation set with various PV panels.

The hotspot phenomenon in local PV panel results from various mechanisms in actual PV applications. For the purpose of guaranteeing the safe operation of the PV modules, the temperature distribution of PV panels should be monitored and predicted for operation and maintenance. The two FBG sensor arrays at an interval of 55 mm were pasted on the front of PV panel with thermal conductive silicone grease and each FBG array had six FBG sensors with the interval of 35 mm that were arranged up and down, as depicted in Figure 4a. The lower end of the fiber was fixed with adhesive tape, and the upper end is in a free state. In this way, the optical fiber can be attached to the photovoltaic panel, and the FBG can be prevented from being affected by the strain deformation of the panel and other stresses. When conducting the outdoor tests, the length of the transmission fiber was 5 m. The six FBGs with different Bragg wavelengths were connected to a transmission fiber in series using the wavelength division multiplexing method. As shown in Figure S16, each spectrum from the two channels of the FBG interrogator displayed the corresponding six Bragg wavelengths from 1530 nm to 1560 nm. The spacing of the two Bragg wavelengths was 5 nm, the bandwidth of the FBG was less than 0.5 nm, and the Bragg grating length was 5 mm. Without an optical gain in the incident light, the reflectivity of each FBG was more than 35%. On account of the sufficient intensity, every Bragg wavelength was demodulated by the peak-seeking algorithm. Before detecting the temperature distribution of a PV panel using FBGs, the temperature sensitivity coefficient of each FBG sensor needed to be calibrated using a temperature control box. According to the calibration results, the relationship of the FBG sensor between the temperature and Bragg wavelength of the FBG sensor was almost linear (Figure S17). The temperature sensitivity levels of the twelve FBGs were close to each other, similar to 10 pm/°C.

The PV panel under the operating condition of power generation was placed outdoors on a sunny day. At the beginning of the experiment, the initial temperature of each FBG sensor was determined by extracting the corresponding temperature of the PV panel from the infrared thermal image at 9:00 a.m. (Figure S18). In this way, the working state of the PV panel and the incidence of hotspots were monitored according to the temperature measurement with the FBG sensor array. Figure 4b demonstrates the hot map collected using the FBG arrays and infrared images of a hotspot on the PV panel; the temperature of the hotspot was 54.5 °C according to FBG and 54.4 °C for thermal imaging. Compared to other areas on the PV panel, the temperature of the hotspot was 10 °C higher than the lowest temperature on the PV panel. The coverage and shadow often have a significant influence on the PV modules, resulting in abnormal working conditions and even producing a hotspot effect. In the experiment, an opaque sticker was employed to cover a part of the P8 cell area, simulating the generation of hotspots. Defects on the PV panels became worse with the increase in the working time; the operating conditions of the PV panel can be evaluated and identified using various artificial intelligence techniques [59,60,61,62]. As shown in Figure 4c–f, the working PV panels were classified into four categories: normal, defective, hotspot and damaged. When the PV panel has an internal defect, the localized temperature is 2~4 °C higher than the rest of the areas under operating conditions (Figure 4d). With shielding on the PV panel, the occurrence of the hotspot effect was determined by a rising temperature of around 10 °C (Figure 4e). Additionally, the long-term hotspot occurred on the PV panel during the period of power generation, an irreversible damage developed with the temperature increasing to 16~18 °C (Figure 4f). After detecting and assessing the PV panels, fast and accurate recognition is required.

Machine learning has emerged rapidly for classification tasks in the field of energy and environment [63]. Fault diagnosis for PV modules can be carried out by applying a machine learning method. A schematic expression of an ANN with classifying PV panels is illustrated in Figure 4g. Considering the temperature field distribution measured with FBG arrays as the input, the forms of a PV panel were the results output using a classifier. The applied ANN with a Bayesian optimization was used to find the optimal architecture of the neural network. Expected improvement was employed as the acquisition function based on the posterior distribution and the next most promising point was selected for objective function evaluation by maximizing the acquisition function. The ANN model has some hyperparameters that need to be tuned, such as the types of activation functions, the number of layers, and the number of nodes. The types of activation functions include ReLU, Tanh and Sigmoid and the number of fully connected layer ranges from 1~3. The number of nodes for each fully connected layer ranges from 1~300. After 1000 training epochs, the optimal architecture of the neural network was carried out with Tanh as an activation function and three fully connected layers which have 124, 4, and 277 nodes separately.

The temperature data, as the input, propagate forward to the hidden layer and then to the output layer. In the outdoor experiment, 2200 temperature field distributions of the PV panel were collected and employed as data samples by training and validating the ANN model. The normal class and defective class have 600 samples, and the hotspot class and damaged class have 500 samples. Five folds of cross validation were performed to protect against overfitting by partitioning the dataset into folds and estimating accuracy on each fold.

Taking advantage of the training model, the working state of the PV panels can be distinguished. The model performance is summarized in a 4 × 4 confusion matrix, as displayed in Figure 4h. From the diagonal elements of the matrix, the 2181 sets were correctly recognized from a total of 2200 samples (19 misclassified samples). In the case of the proposed classifier, a classification rate of 99.1% was achieved after 1000 model training epochs. Except for the ANN model, various other classifiers were used for the comparison, including the decision tree model, the support vector machine (SVM) model, the k-nearest neighbor (KNN) model and the one-dimensional convolution neural network model (1-D CNN). Applying a classifier of the decision tree model, 54 samples were misclassified out of 2200 samples with an accuracy of 97.5% (Figure S19a). When evaluating the SVM classifier, 68 samples were misclassified from all samples, leading to a classification rate of 96.9% (Figure S19b). With the KNN model, 74 samples were wrongly classified from 2200 samples with a precision of 96.6% (Figure S19c). Considering the 1-D CNN model based on deep learning, 28 samples were wrongly classified from 2200 samples with a precision of 98.7% (Figure S19d). Accordingly, the ANN classifier outperformed all of the compared classifiers. Similarly, the receiver operating characteristic (ROC) is a metric used to check the quality of classifiers. The area under a ROC curve (AUC) provides an aggregate performance measure across all possible thresholds; the AUC values are in the range [0, 1] and larger values indicate better classifier performance. As presented in Figure S20, the AUC values of four categories were both close to 1 and the hotspot and damaged AUC values were slightly lower the other values because the temperature of the two types of PV panels is frequently affected by the climate resulting in the difficulties in identification.

In terms of hotspots, it is worthwhile for solar PV modules to be maintained in advance, improving the power generation and extending the life span. In view of the recognition rate of a classifier model, the trained ANN model trained was applied to distinguish between the working conditions of a PV panel. When the hotspot had been identified by the proposed classifier, a cooling hydrogel was used to lower the temperature of the hotspot. Figure S21 shows the hot map of the PV panel after cooling using the PAM-CaCl_2_ hydrogel, shown in Figure 4b. The temperature of the hot spot area is 7.7 °C lower than that before cooling. Even if the PV panels with hotspots cannot be returned to the factory for repair or continue to be used, safety hazards can be avoided by employing in this approach. Moreover, in order to evaluate the durability of the PAM-CaCl_2_ hydrogel, a seven-day outdoor test was also conducted in our work from 5 July to 7 July 2024, and from 29 July to 1 August 2024. There was no significant change due to the influence of the environment in the thermal silicone grease after seven-day outdoor testing, meaning that the experimental results were not affected. The PAM-CaCl_2_ hydrogel demonstrated consistent cooling ability under repeated sorption–evaporation cycles, as shown in Figure S22. In the seven-day test, the PAM-CaCl_2_ hydrogels achieved a daytime cooling effect of 2~14 °C and the temperature difference in the whole daytime range (6:00~18:00) was 5.9 °C when compared to the normal PV panel. The PAM-CaCl_2_ hydrogel was exposed for almost one month and only partially upwarped with no salt leakage, as seen in Figure S23.

### 2.5. Field Tests of Cooling and Power Generation Performance

**Figure 5:**
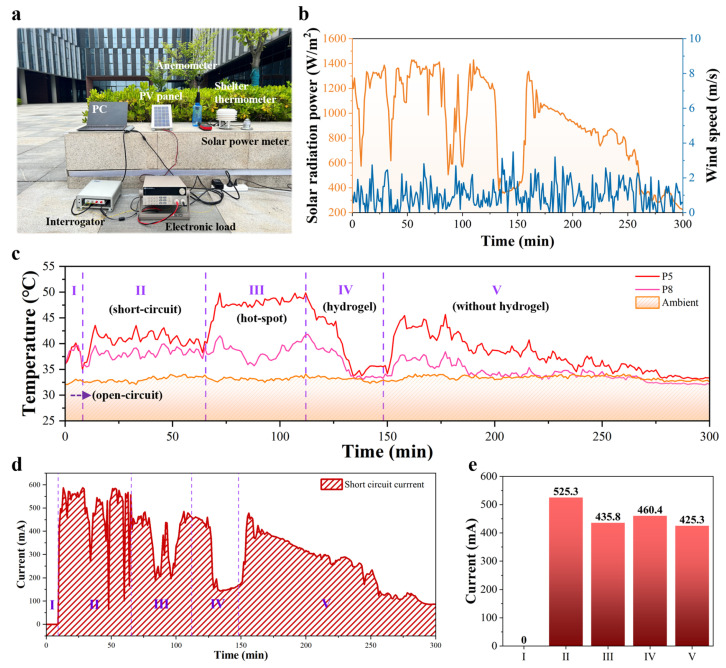
Field tests of the temperature and current of the PV panel with a hot spot. (**a**) Photograph of the outdoor field test setup in Nanjing University campus. (**b**) The solar radiation power and wind speed from environmental conditions. (**c**) 5-h temperature profile measured of the PV panel with the ambient temperature on 18 June 2024. (**d**) The short-circuit current measured of the PV panel corresponding to (**c**). (**e**) Comparison of currents of the PV panel between different stages under the similar sunlight.

In order to study the cooling and power generation of the PV module with a hotspot on the relevant time scale, field tests were performed with a period of five hours divided into five stages. As displayed in Figure 5a, the outdoor experimental setup included a PV panel, two FBG sensor arrays, a cooling hydrogel, an FBG interrogator for demodulating FBGs, an electronic load for recording power generation, a notebook computer for collecting various data, an anemometer, a shelter thermometer and a solar power meter (see details in Note S1). The two FBG arrays with six temperature sensors each were fixed on the surface of the PV panel for temperature detection. When the hotspots were diagnosed by applying the proposed method, a prepared PAM-CaCl_2_ hydrogel for cooling was pasted on the back of the region of hotspots. Figure S24 demonstrates the photograph of the PV panel’s backside with a cooling hydrogel attached, which had a size of 5 × 5 cm^2^ and 5 mm thickness. Due to the uniformity of sunlight irradiation and thermal convection, a PV panel was placed with a tilt angle of 45° using a bracket.

The field tests took place in June because the outdoor environment had enough solar radiation power and high temperatures, which easily cause safety hazards. As depicted in Figure 5b, the local climate, including solar irradiance and wind speed, was recorded from 11:25 to 16:25 on 18 June 2024. The maximum solar radiation power of the day reached up to 1400 W/m^2^ and the range of wind speed ranged from 0 m/s to 3 m/s. There were five stages in the process of the outdoor field test. The temperature curve and the corresponding current curve were divided into five parts, as described in Figure 5c,d, respectively. As for the study of hotspots, the temperature of the non-hotspot area (P8 region) on the PV panel was compared with the high temperature of the hotspot area (P5 region). Considering the influence of the environmental temperature, the ambient temperature of the test environment was recorded as ranging from 32 °C to 34 °C using a shelter thermometer. Allowing for the generation of hotspots, the loop current in the PV panel should be sufficient. Thus, the electronic load was set to zero and the short-circuit current (SCC), as a power generation parameter, was recorded in real time (Figure 5d). The sampling rate was set to once per second for each test instrument. In region I, the current was always zero with the PV panel in an open-circuit state. Meanwhile, the temperatures measured for P5 and P8 were almost the same and 7 °C higher than the ambient temperature. Instead of the open-circuit state, the temperature detected for P5 and P8 in region II of the short-circuit state had increased widely and the temperature became uneven during the whole of second stage. The difference between P5 and P8 was 2.6 °C, a difference caused by the cell of P5, which was an internal defect in the PV panel, as shown in Figure S25. In parallel, the SCC was changed from 63.9 mA to 587.9 mA under the variable solar radiation and the mean SCC was 499.4 mA. In region III, an opaque sticker was pasted on the PV panel to cover 20% of the P5 cell area by simulating the hotspot effect. The gap between P5 and P8 was 9.1 °C, approximately, resulting in a clear drop with the mean of 100 mA in SCC. The peak value of the P5 temperature reached 50 °C within six minutes from 40.1 °C, indicating the rapid generation of hotspots in a short period of shading. Thus, it is necessary for PV modules to be subjected to a quick hotspot diagnosis. When a cooling hydrogel was attached to the back of the hotspot area, the temperature of P5 was decreased from 49.8 °C to 33.8 °C with 22 min via cooling due to the hydrogel and wind in region IV. Considering only the cooling effect of the PAM-CaCl_2_ hydrogel, the difference in the temperature change between P5 and P8 had the value of 7.7 °C. Unfortunately, the solar radiation power appeared in a wide valley in the fourth stage, leading to an incomprehensible SCC changes due to cooling. In the last region, the cooling hydrogel was removed, with the P5 temperature rising again. However, the P5 temperature continually fell from 45.6 °C to the ambient temperature in fifth stage, with a persistent decrease in the solar radiation power. At the same time, the difference in temperature of P5 and P8 was 6.4 °C. Like region IV, the trend in SCC was consistent with the variation in the solar radiation power. To exclude the effect of sunlight on SCC, the comparison of the five stages can be organized under the same solar radiation power. As shown in Figure 5e, the SCC comparison of the five regions was carried out under a similar solar radiation power, with 1.2 kW/m^2^. At the beginning of the first region, SCC was zero due to an open-circuit state. In region II, SCC reached up to 525.3 mA with a short-circuit state. With a shading in region III, SCC decreased to 435.8 mA by 17% and then rose up a little to 460.4 mA by 5.6% after cooling in region IV. Finally, SCC also declined to 425.3 mA when removing the cooling hydrogel. Through carrying out a hotspot management system, the hotspot can be detected and cooled down by realizing predictive maintenance.

As seen in Table 1, the proposed thermal management system can achieve high classification accuracy, an ideal cooling effect and improved power generation efficiency. Compared with infrared images with machine learning, the suggested system identifies hotspots with over 95% accuracy. In terms of the cooling effect, hydrogels are not as effective as liquid cooling, but they do not consume extra energy. In addition, the hotspot effect of PV modules was successfully recognized and inhibited using the proposed hotspot management system integrated with the FBG temperature sensor array and cooling hydrogel.

## 3. Conclusions

The hotspot effect of PV modules during the spatial–temporal operation was successfully discovered and inhibited using the proposed hotspot management system integrated with FBG temperature sensor arrays and PAM-CaCl_2_ cooling hydrogels. Based on a three-dimensional finite element simulation, the results revealed the temperature model of a PV cell obtained according to environmental factors including solar radiation power, ambient temperature, and wind speed, as well as the cooling effect of 11.8 °C by a PAM-CaCl_2_ hydrogel. The cooling effect measured using the FBG sensors was in line with the simulation; additionally, the gain in power generation was confirmed through indoor experiments. The outdoor field tests performed in the summer showed that the optimized ANN classifier evaluated the hotspots of PV panels with an accuracy of 99.1% by monitoring the temperature distribution with FBG arrays; then, a cooling effect of about 7.7 °C with a PAM-CaCl_2_ hydrogel was realized as feedback. The dynamic evolution of hotspots revealed that a hotspot with a temperature increase of 9 °C was created rapidly in 6 min and cooled down to ambient temperature within 22 min. With the hotspot effect, the short-circuit current of the PV panel was dropped by 17% and raised by 5.6% after cooling. The suggested hotspot management strategy provides valuable insights for predictive maintenance in the event of hotspots. In the future, hotspots should be diagnosed early and cooled effectively to avoid safety hazards, extend the lifetime of PV modules and improve the power generation efficiency.

## Figures and Tables

**Table 1:** Comparisons of thermal management system.

Detection/Cooling Methods	Classification Accuracy	Temperature Reduction	Improved Efficiency
Infrared images with a Naive Bayes classifier [59]	94.1%	/	/
Infrared images with a support vector machine classifier [62]	92%	/	/
Air cooling with aluminum fins [24]	/	7.4 °C	2.72%
Liquid cooling with pulsating heat pipes [26]	/	16.1 °C	18%
This study	99.1%	7.7 °C	5.6%

## Data Availability

Data will be made available on request.

## Supplementary Materials

The following supporting information can be downloaded at: https://www.mdpi.com/article/10.3390/s25154879/s1, Note S1: Supplemental Experimental Methods and Supporting Figures: Figures S1–S25.
