# Supplementary material for: Spatial–Temporal Hotspot Management of Photovoltaic Modules Based on Fiber Bragg Grating Sensor Arrays
Source: Sensors (Basel). 2025 Aug 7;25(15):4879. doi: 10.3390/s25154879 (PMC12349028; doi:10.3390/s25154879)
Supplement: Supplementary file 1 [file sensors-25-04879-s001.zip › sensors-3790214-supplementary.pdf]

Supplementary information for

Spatial–Temporal Hotspot Management of Photovoltaic Modules Based on Fiber Bragg Grating Sensor Arrays

Haotian Ding [1,†], Rui Guo [1,†], Huan Xing [1], Yu Chen [1], Jiajun He [1], Junxian Luo [1], Maojie Chen [1], Ye Chen [2,*], Shaochun Tang [1,3,*] and Fei Xu [1,*]

[1] Key National Laboratory of Solid State Microstructures, Collaborative Innovation Center of Advanced Microstructures, College of Engineering and Applied Sciences, Nanjing University, Nanjing 210023, China
[2] College of Physics, Nanjing University of Aeronautics and Astronautics, Nanjing 210016, China
[3] Haian Institute of High-Tech Research, Nanjing University, Nanjing 226600, China
* Correspondence: yechen@nuaa.edu.cn (Y.C.); tangsc@nju.edu.cn (S.T.); feixu@nju.edu.cn (F.X.)
† These authors contributed equally to this work.

This PDF file includes:

Supporting Notes

Note S1. Supplemental Experimental Methods

Supporting Figures: Figures S1–S13

Note S1. Supplemental Experimental Methods

Fabrication of FBG sensor arrays

The FBG sensor arrays were inscribed with a femtosecond laser (Pharos, Light-conversion) and a three-dimensional precision displacement stage (Physik Instrument). The fs laser with a central wavelength of 1028 nm and a pulse width of ~290 fs was set at 1 kHz repetition rate. Firstly, a single-mode fiber (Corning SMF-28) was held on the three-dimensional displacement stage that can be moved along the axis of the stage. Then the laser beam was focused by a 100× oil objective (Olympus, NA = 1.30). During the fs FBG inscription, the laser beam was focused on the inner cladding of the SMF with pulse energy of ~300 nJ. The velocity of the stage varied from 1.0620 mm/s to 1.0795 mm/s with the interval of 0.0035 mm/s, which correspond to the 2nd-order grating wavelength from 1535 nm to 1560 nm. FBGs were monitored by an optical analyzer (Yokogawa-AQ6370C) to judge the sensors performance.

Materials

N, N, N'N'-tetramethylenediamine (TEMED) was purchased from Shanghai Maclin Biochemical Technology Co. LTD. Calcium chloride and potassium persulfate (KBS) were both purchased from Mairel Experimental Equipment (Shanghai) Co., LTD. Acrylamide monomer (AM) was purchased from Tixiai (Shanghai) Chemical Industry Development Co., LTD. N,N' -methylene bisacrylamide (MBA) was purchased from Anhui Zesheng Technology Co., LTD. All chemicals were used directly without further purification.

Synthesis of PAM-$CaCl_2$ hydrogel

In a classic method, 5.33 g acrylamide monomer was fully dissolved in 43 ml deionized water under nitrogen atmosphere. 0.032 g N,N'-methylene bisacrylamide as a crosslinker and 40 µl N, N, N'N' -tetramethylenediamine as accelerator were added and stirred. Then 0.064 g potassium persulfate was added as an initiator and the solution was crosslinked in an oven at 50°C for 2 hours. After drying at 80 °C for 10 hours, the dried hydrogel was immersed in 125 ml calcium chloride solution (0.4g/ml), which left for 8 hours and then placed in the indoor environment condition for one day to obtain the PAM-$CaCl_2$ hydrogel.

Finite element simulation

To investigate the temperature field of a PV cell, three-dimensional COMSOL Multiphysics simulator was applied to solve the corresponding research. The length, width and height of the total solar cell model were set to 5 cm, 5 cm, 2.14 cm and the hydrogel thickness was set to 1 cm. In the construction of the temperature field, heat transfer module and radiation module were applied and integrated to set up environmental conditions. A finite-element-method (FEM) analysis was carried out. There were two studies in the simulation, such as the influence of the relevant influencing factors on the temperature field of a PV cell and the cooling effect of a hydrogel for the PV cell. The mesh setting was used as refinement and the time spent on the study was about 32 hours and 9.5 minutes respectively.

Optical measurement

The optical signals were acquired by the interrogator from Smartfiber Technology ltd with the spectral range from 1528.670 nm to 1568.470 nm and a sampling interval of 100 pm. Through a fitting algorithm, the wavelength resolution of the interrogator is 1 pm. The sampling rate of the interrogator was set to 1 Hz in the measurements.

Temperature calibration of FBG sensors

The temperature sensitivity measurements were performed using a temperature control box. The temperature of the control box was set from 30 to 80 °C. The Bragg wavelengths of the FBGs were recorded for each 10 °C which was maintained for 60 s to average at each level.

Electrical measurement

For the electrical tests, the indoor polycrystalline PV cells (50×50×2 mm) with the maximum power of 0.5 W were purchased from Juyang Technology Electronics (Shenzhen) Co., Ltd. and the outdoor polycrystalline PV panels (245×145×17 mm) with the maximum power of 3 W were purchased from Foshan Aike Electronics Engineering Co., Ltd. Indoor PV electrical data including I-V curves, V_{oc} and P_{max} were measured using an electrochemical workstation (PGSTAT302N, Autolab). The short-circuit current measured of the outdoor tests was realized by a DC electronic load (IT8510) from Itech Electronics Co., LTD with the detection resolution of 0.1 mV/1mA and the measurement accuracy is ±0.05%.

Classifier based on machine learning

All classifiers selected were derived from the classification learner application in MATLAB 2023a. Confusion matrix and the area under a ROC curve (AUC) were chosen to evaluate the recognition accuracy and precision of multiple classifiers with various categories.

Outdoor field tests

Field tests were conducted on the campus of Nanjing University (32°7′N 118°56′E). The PV temperature was measured by FBG sensor arrays with aid of the infrared thermal imaging system (FOTRIC 320, Testo). The solar irradiation during the tests was analyzed by a solar power meter pyranometer (RS-RA-N01-AL), the wind speed was recorded by an anemometer (GM8907, Benetech) and the ambient temperature was measured by a shelter thermometer (RS-BYH-M). The measurement accuracy of the solar radiation, wind speed, and ambient temperature was, ±1 W/m^2, ±0.1 m/s, and ±0.5 °C, respectively.

Supporting Figures

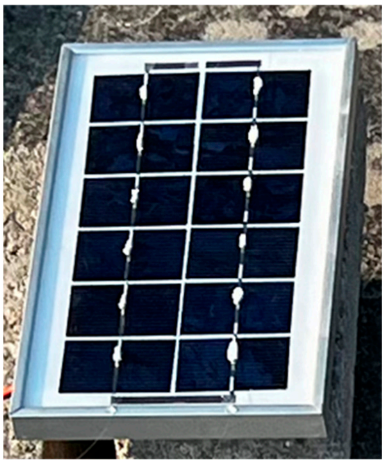

Figure S1. The photograph of the FBG arrays attached to the photovoltaic panel with thermal conductive silicone grease.

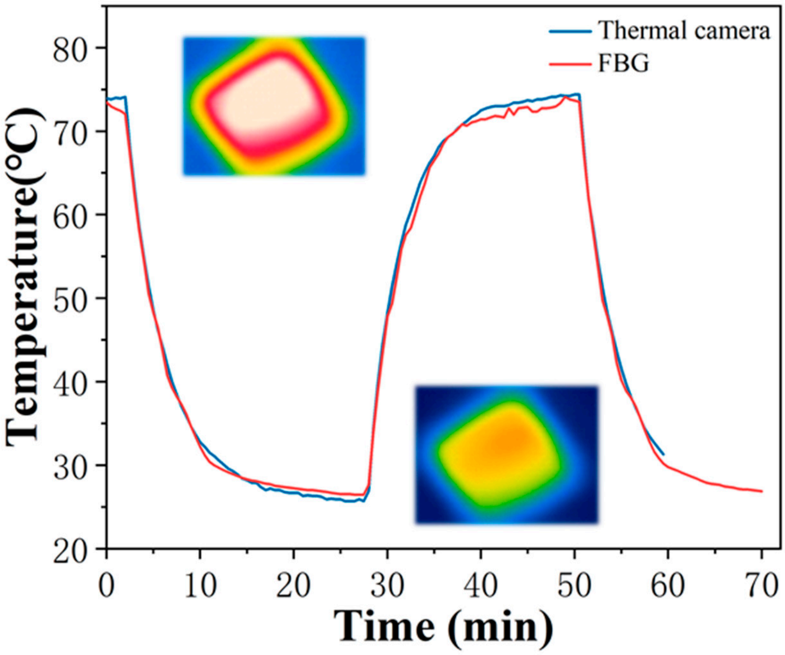

Figure S2. Comparison of temperature measurement between FBG and thermal camera. As a result, the temperature measurement accuracy of FBG is consistent with thermal imaging.

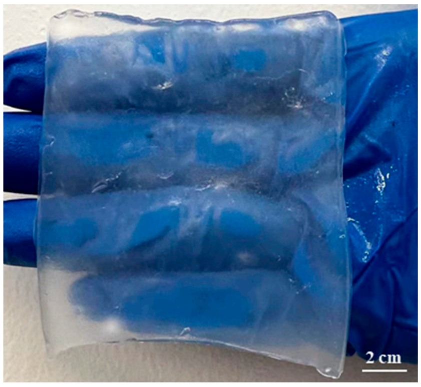

Figure S3. A digital photo of the hygroscopic PAM-$CaCl_2$ hydrogel.

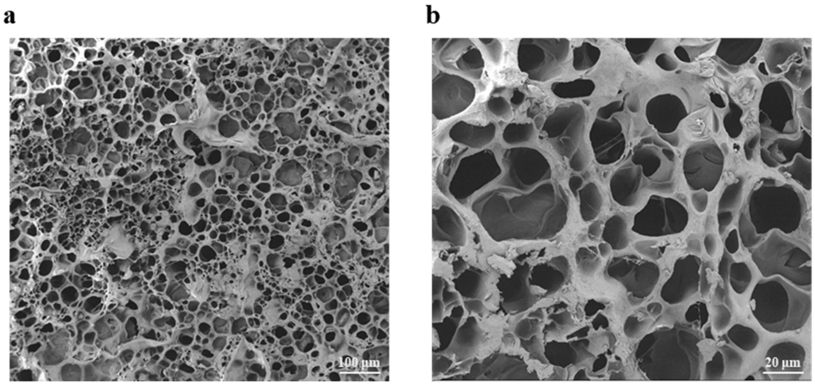

Figure S4. SEM image (a) of the hygroscopic hydrogel and magnified SEM image (b) of the freeze-dried PAM-$CaCl_2$ hydrogel.

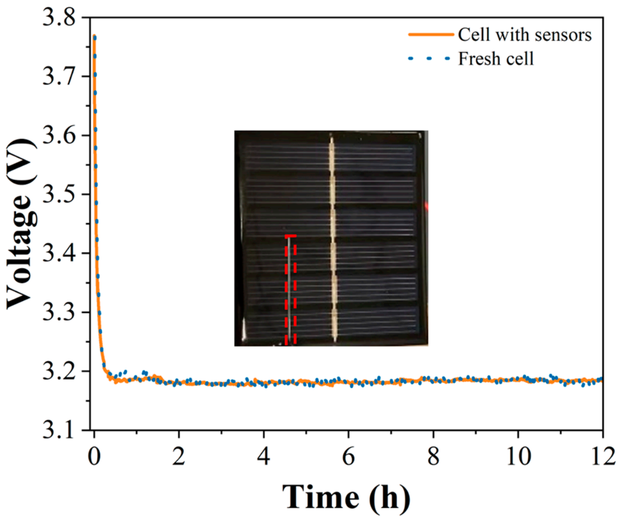

Figure S5. The cell performance of solar cells attached with FBG and without FBG. The insert figure shows a solar cell attached with FBG.

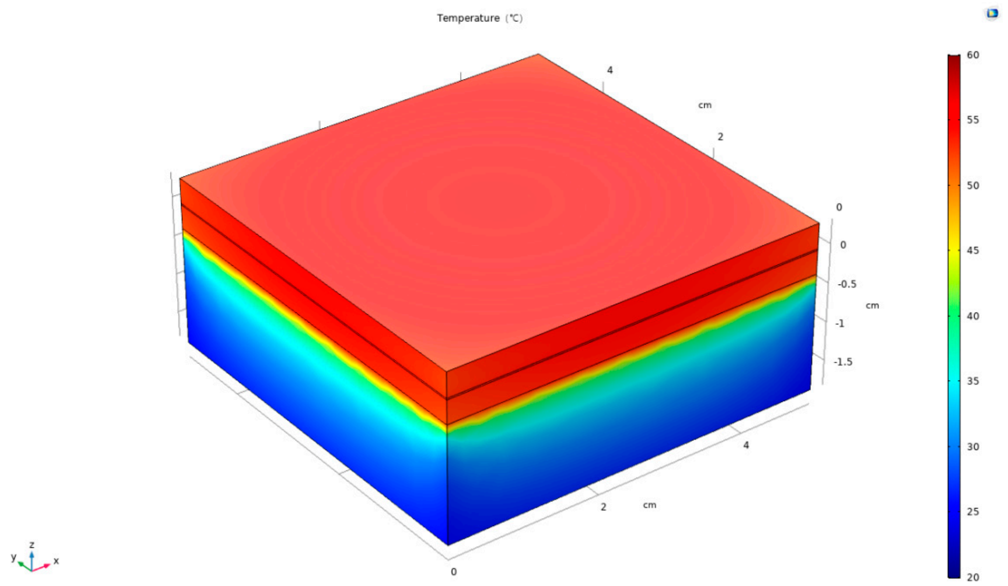

Figure S6. The temperature model of a photovoltaic panel under the condition of one sun radiation, room temperature of 25 °C and natural thermal convection.

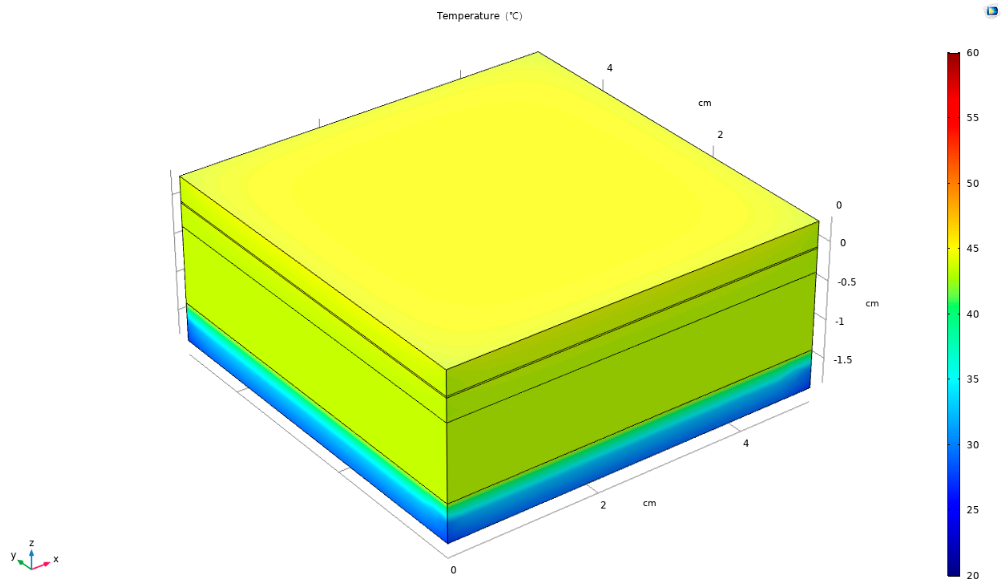

Figure S7. The temperature simulation of a photovoltaic panel with a cooling hydrogel under the same condition of Figure. S6.

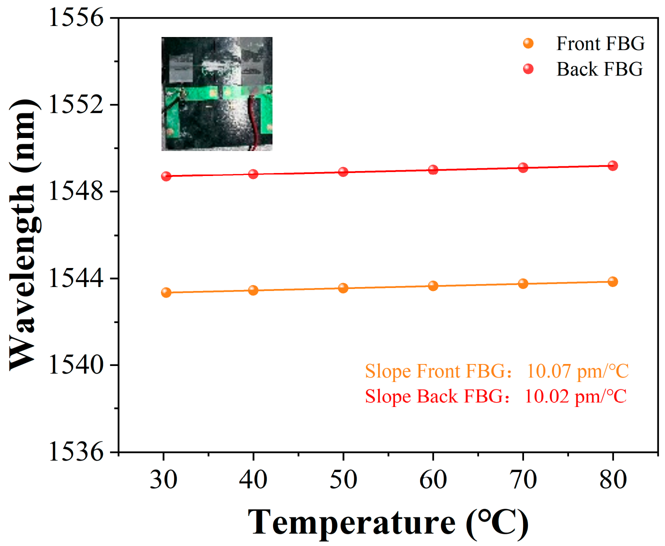

Figure S8. The temperature calibration of two FBG sensors, which one FBG is on the surface of a photovoltaic cell and the other on the back. The points are linearly fitted by two lines. The linear relationships with the two slopes are given. The insert figure shows an FBG pasted on the back of a solar cell with thermal conductive silicone grease.

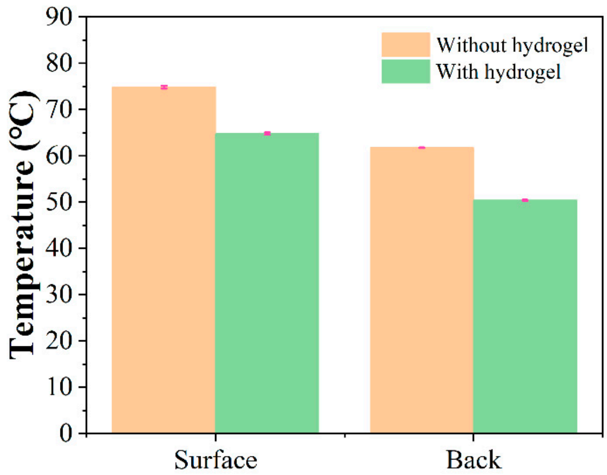

Figure S9. The surface and back temperature by a bare solar cell and a solar cell with the cooling hydrogel.

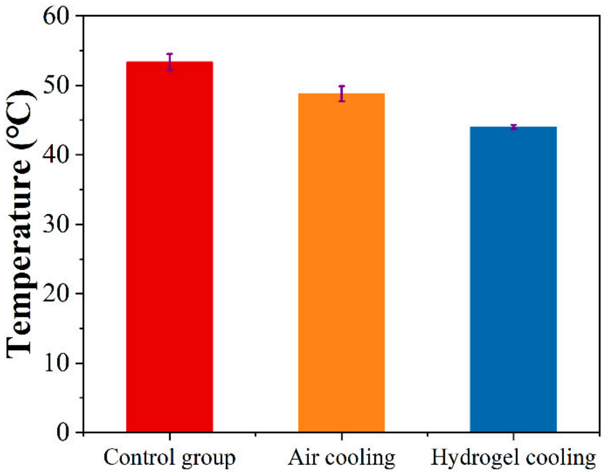

Figure S10. Average temperature and flux of solar cells between natural convection, air cooling and hydrogel.

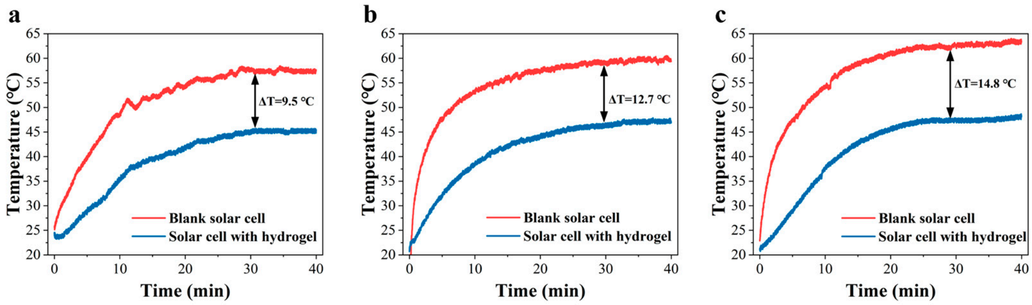

Figure S11. Cooling performance of the hydrogel with different solar intensity under (a) 0.8 sun, (b) 1 sun, and (c) 1.2 sun.

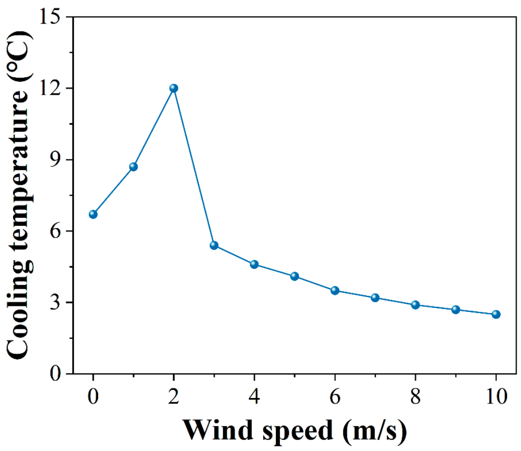

Figure S12. Simulation of cooling performance with the hydrogels under varying wind speeds.

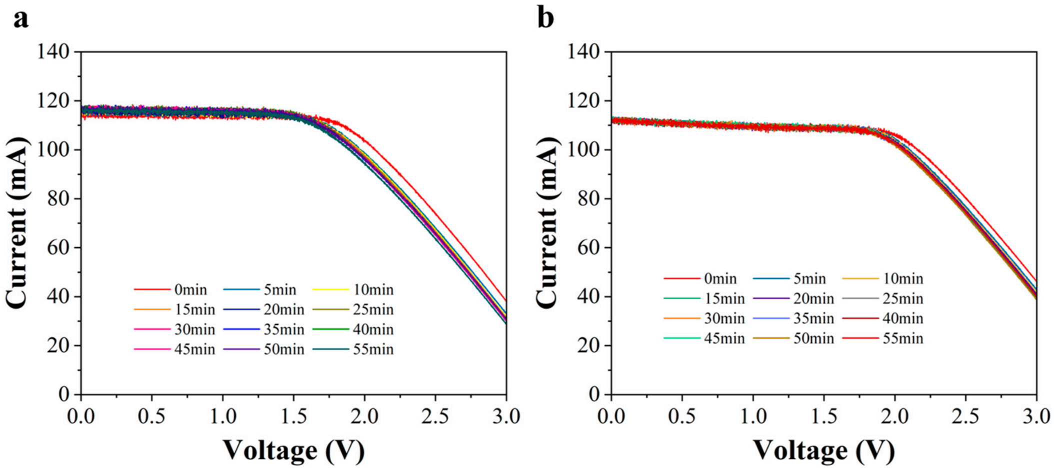

Figure S13. I-V curves of PV cell (a) without the cooling hydrogel and (b) with the cooling hydrogel under 1 kW/m^2 sunlight irradiation.

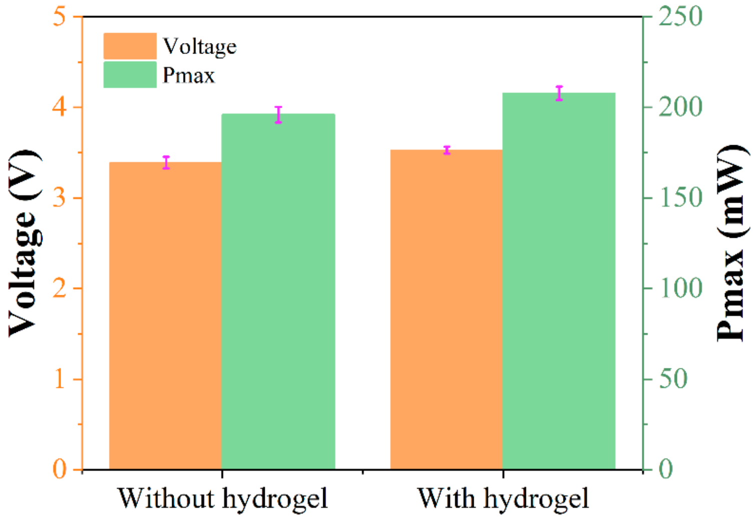

Figure S14. The open circuit voltage (V_{oc}) and maximum power (P_{max}) of a solar cell with and without the hydrogel cooling layer.

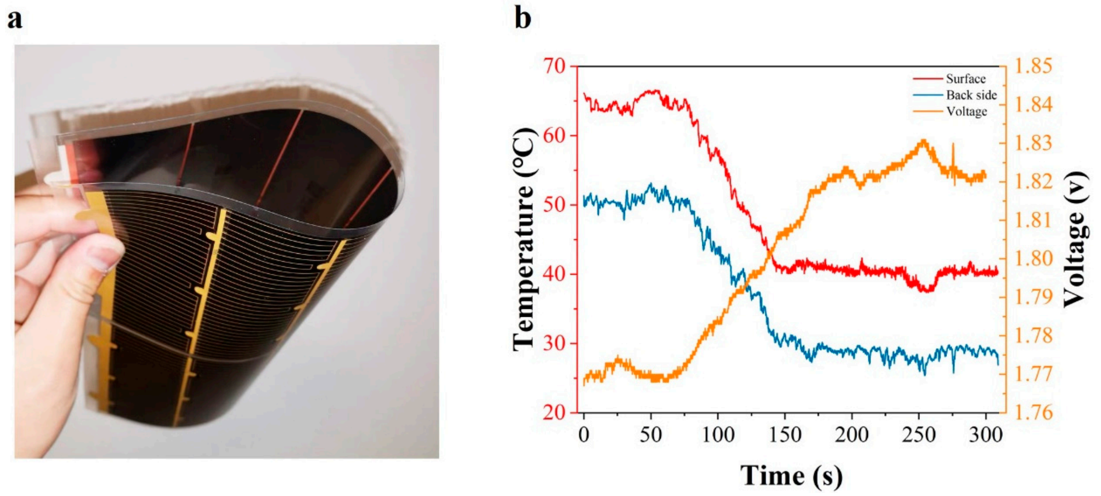

Figure S15. (a) A digital photo of the flexible solar cell with copper indium gallium selenide. (b) The cooling effect and cell improved performance of the temperature control system applied in the flexible solar cell.

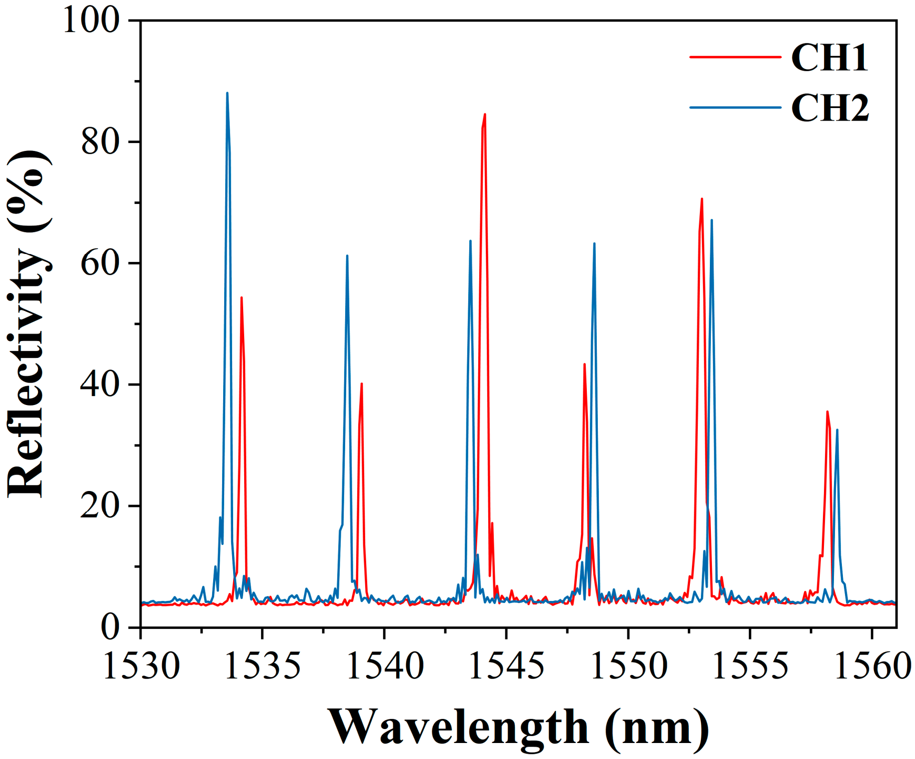

Figure S16. The spectrum of two FBG sensor strings.

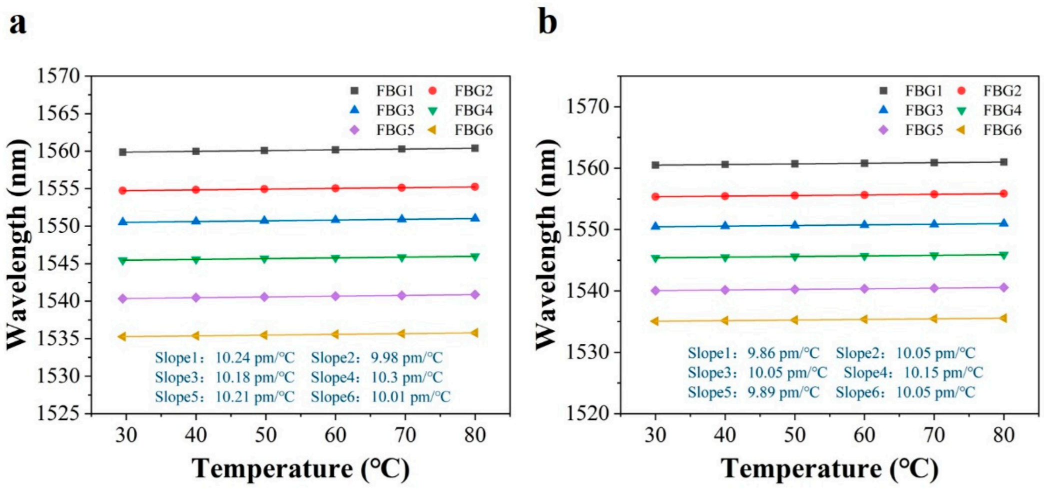

Figure S17. The temperature calibration of two FBG sensor arrays with 6 FBGs. The linear relationships with each fitting slope are given, are both similar to 10 pm/°C.

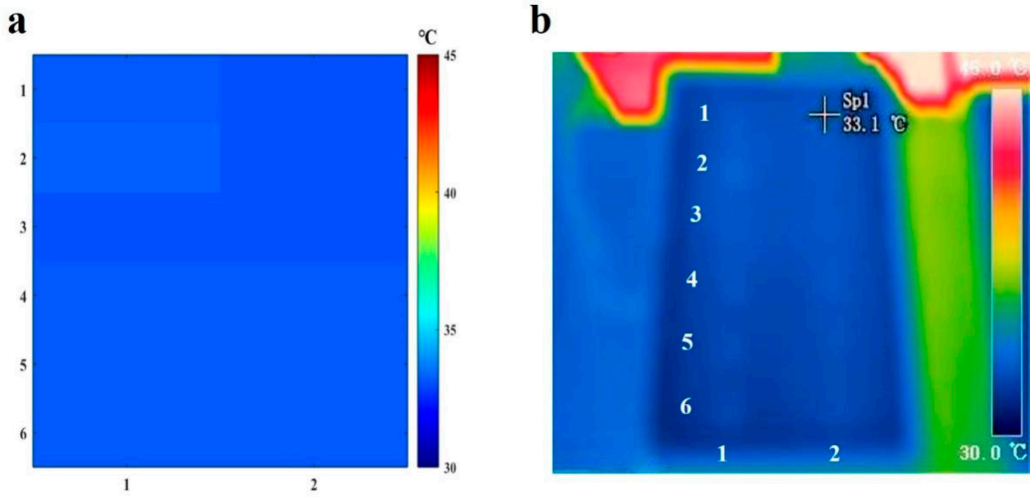

Figure S18. (a) The hot map by FBG arrays and (b) infrared thermal image of the photovoltaic panel at 9:00 a.m. Extracting the corresponding temperature of the photovoltaic panel from infrared thermal image as the initial temperature of each FBG, which is about 33 °C.

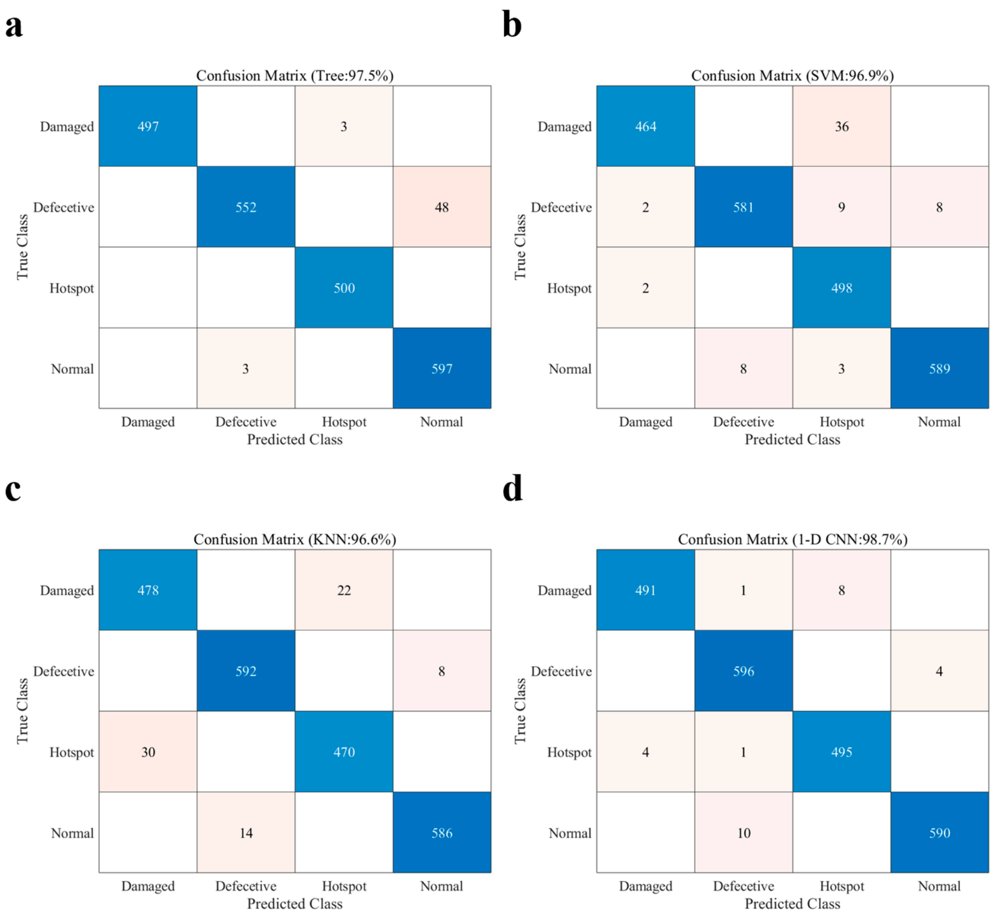

Figure S19. Comparison of different classifiers in machine learning. The Confusion matrix of (a) the decision tree model, (b) the support vector machine model, (c) the k-nearest neighbor model and (d) the one-dimensional convolution neural network model.

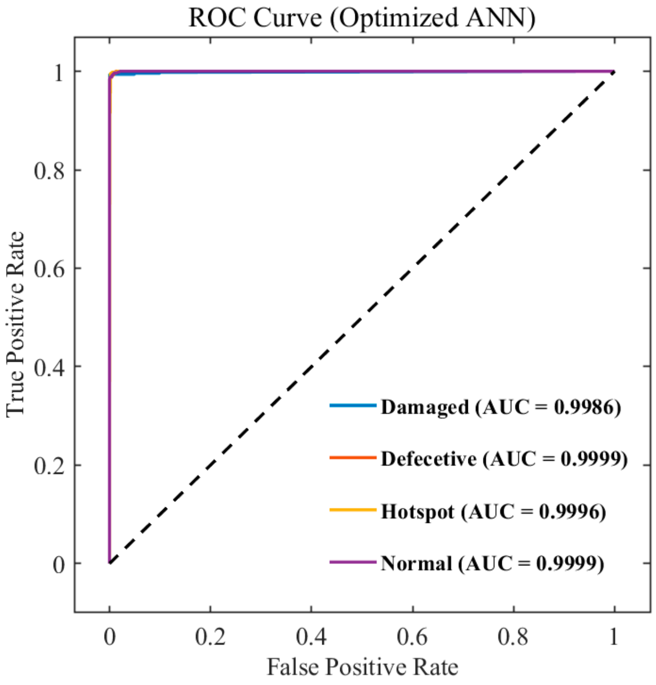

Figure S20. ROC curves of four categories of PV panels.

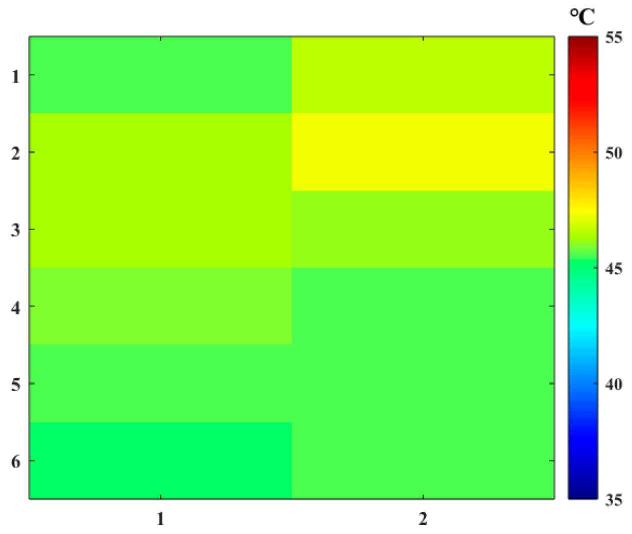

Figure S21. The hot map of the photovoltaic panel after cooling by PAM-$CaCl_2$ hydrogel. The temperature of the hot spot area is 7.2 °C lower than that before cooling.

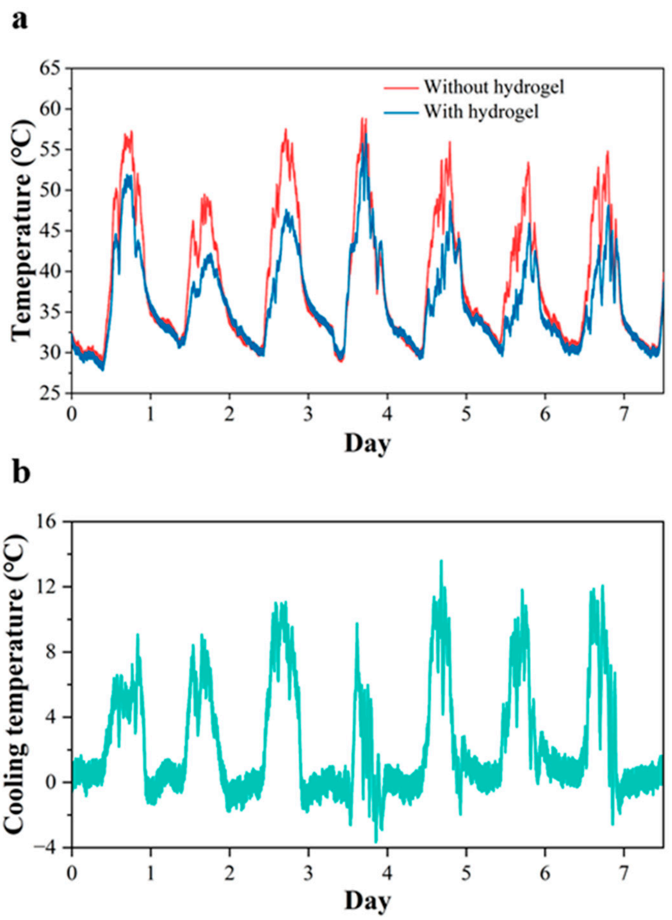

Figure S22. 7-day outdoor testing of (a) temperatures for two PV panels and (b) cooling temperature.

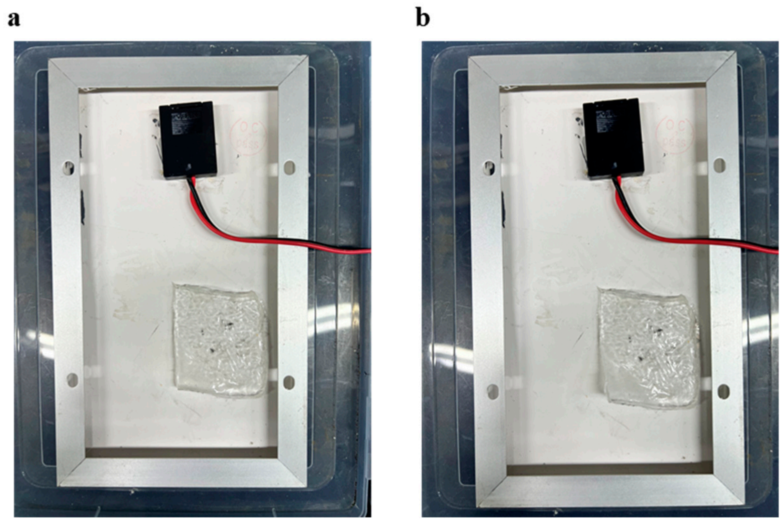

Figure S23. Comparison of PAM-$CaCl_2$ hydrogel application for one month. (a) Original PAM-$CaCl_2$ hydrogel. (b) PAM-$CaCl_2$ hydrogel after using.

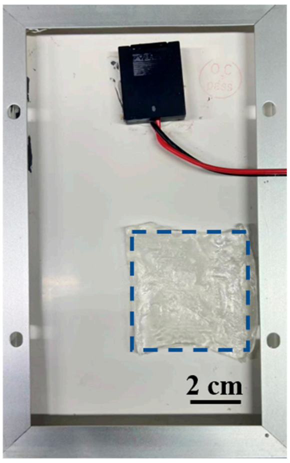

Figure S24. Digital photo of the PV panel backside with a cooling hydrogel.

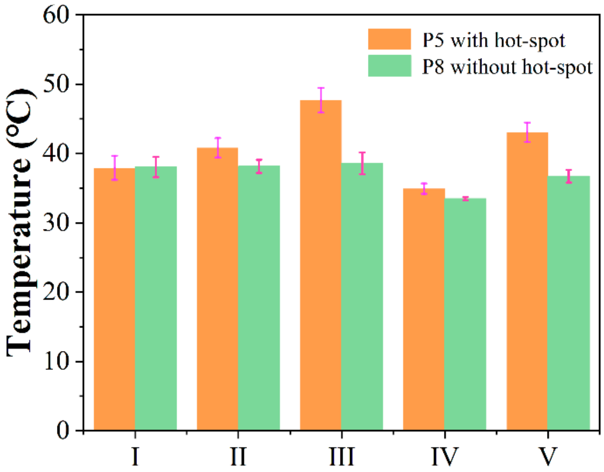

Figure S25. P5 and P8 measured temperature of the PV panel within five stages.
